# *Dendrophthoe pentandra* (L.) Miq extract effectively inhibits inflammation, proliferation and induces p53 expression on colitis-associated colon cancer

**DOI:** 10.1186/s12906-016-1345-0

**Published:** 2016-09-26

**Authors:** Agustina Tri Endharti, Adisti Wulandari, Anik Listyana, Eviana Norahmawati, Sofy Permana

**Affiliations:** 1 Department of Parasitology, Faculty of Medicine, Brawijaya University, Malang, Indonesia; 2 Master Program in Biomedical Science, Faculty of Medicine, Brawijaya University, Malang, Indonesia; 3 Department of Anatomical Pathology, Faculty of Medicine, Brawijaya University, Malang, Indonesia; 4 Department of Biology, Faculty of Mathematics and Natural Sciences, Brawijaya University, Malang, Indonesia

**Keywords:** *Dendrophtoe pentandra*, IL-22, MPO, Proliferation, p53, CAC

## Abstract

**Background:**

Indonesian mistletoe grows on various trees. Mango Mistletoes *(Dendrophthoe pentandra)* is one type of mistletoe that grown on mango tree (.benalu mangga in bahasa Indonesia). Our study used mistletoe as a parasitic plant that has been used for traditional medicine. It has been known that *Dendrophtoe pentandra* extract (DPE) anti-inflammatory and anticancer. Furthermore, it is necessary to follow-up study in vivo to evaluate the response to treatment of new cancer therapeutic agents. This research aimed to determine the levels of IL-22, myeloperoxide (MPO), proliferation and wild-type p53 expression after the administration of DPE to murine models of CAC.

**Methods:**

Mouse colitis associated colon cancer (CAC) was induced firstly by azoxymethane (AOM) and followed by administration of drinking water containing 5 % dextran sodium sulfate (DSS) in a cycle protocol, each cycle consisted of seven days of 5 % DSS in the drinking water and followed by seven days of regular water. This study consists of five treatment groups: I was treated water only (control), II was administrated by (DSS only, without DPE), (III-V) were administrated by DPE (125 mg/kg BW, 250 mg/kg BW and 500 mg/kg BW) respectively. The administrated of DPE were started from the 8^th^ weeks, were continued until 21 weeks. At the end of 21 weeks of the experiment, mice were sacrificed, colon tissue was removed, and then subjected to ELISA, flow cytometry, real-time PCR and histology examination.

**Results:**

Administration of DPE 250 mg/kgBW significantly reduce the levels of IL-22 and MPO compared with DSS only group (*p* < 0.001; *p* < 0.001). Colonic epithelial cells proliferation of group IV (DPE 250 mg/kgBW) were significantly lower than III and V groups. There was no significant change in the S phase in mice were treated DPE 125 mg/kg BW and 500 mg/kg BW, while administration of DPE 250 mg/kg BW was able to increase the percentage of cells in S phase. The expression of mRNA p53 was up regulated in mice received DPE 125 mg/kg BW.

**Conclusion:**

These findings indicate that the DPE could inhibit colonic epithelial cells proliferation through p53 pathway independently. This study also showed that DPE could be potential sources of new therapy.

## Background

Since mistletoe is a semi-parasitic plant, it is suggests that their bioactivities could also depend on their host plant [1]. However people in Indonesia usually called the mistletoe depend on the host plant where it grew, such as benalu mangga (mistletoe which used mango as host).

Mistletoe is considers as an unwanted plant to economically important horticultural plant, however in the other side, mistletoe is known as one of medicinal plant used in traditional/alternative medicine in Indonesia and other countries such as in treatment for cough, diabetes, hypertension, cancer, diuretic.

DPE contains quercetin-3-rhamnose [2]. Quercetin has anti-oxidant, anti-inflammatory, anti-cancer, and immune modulator effect [3–5]. Quercetin also improve the action of the drug 5-Fluorouracil (5-FU) promoting increased expression of p53 and apoptosis in breast cancer T47D cells [6, 7].

Colon cancer is caused by many factors that affect multiple etiological pathways. Important risk factors include chronic inflammation, environmental effects and unhealthy lifestyle. All these risk factors are linked to cancer through inflammation [8].

CAC is modeled in mice by administering AOM and DSS. The combined administration of AOM and DSS accelerates tumor growth [9]. The induction AOM and DSS in Balb/C mice lead to 100 % development of adenocarcinoma of the colon It also mimics inflammatory reactions seen in patients with ulceratif colitis (UC) [10]. Chronic inflammation in mice model that are initiated by the DSS causes colitis, deoxyribonucleic acid (DNA) damage and encourages the emergence of adenoma [11, 12]. In CAC, intestinal inflammation play critical role that induce DNA damage and cellular proliferation pathway. That will lead CAC by increasing of cell signals, cell proliferation and suppressing of tumor suppressor and apoptosis [13, 14]. This CAC is associated with the cell cycle and its regulation is affected by the tumor suppressor proteins.

One of the tumor suppressor proteins such as p53 play an important role in the cell cycle and apoptosis. The function of p53 instead a transcription factor, targeting several genes that play a role in the cell cycle checkpoint and induction of apoptosis [15, 16].

Chronic inflammation causes the migration of inflammatory cells such as neutrophils. If the production of neutrophils is excessive, it will cause damage to tissue [17]. Interleukin 22 is a cytokine that is induced by cytokines IL-6, IL-23, IL-1β and TNF-α under conditions of inflammation in response to tissue damage. Interleukin-22 binds IL-22R1 receptor complex, activates STAT3 [18] and encourage tissue repair to improve colonic epithelial cell proliferation in acute inflammatory conditions [19]. Interleukin-22 is deregulated in cancer conditions, tissue regeneration regulation function is transformed into the driving cancer development [20].

There is no information regarding the influence of DPE treatment on CAC model. The mechanism of DPE as anti inflammation and proliferation still unknown. Hence we investigate the effect of DPE on the levels of IL-22, myeloperoxide (MPO), proliferation, and expression of wild-type p53 following DPE administration to murine models of CAC.

## Methods

### Plant material and extraction

Mango Mistletoes *Dendrophthoe pentandra* leaves were collected from Probolinggo, East-Jawa, Indonesia. The plant was identified and authenticated by biologists in Department of Biology, University of Brawijaya (specimen No.0170/Taxonomy Identification/03/2015). The leaves were dried for five days at room temperature and then powdered. Leaves were macerated with 80 % ethanol for 72 h and then filtered. The filtrated was evaporated under reduced pressure to obtain of solid form of the extract. The quercetin content from DPE extraction was 0.57 μg/g dry weight that quantified by thin layer chromatography (TLC).

### Mice

Female Balb/c mice, aged 8–10 weeks, weight between 20–25 g were maintained in a relative humidity of 50–55 % and a preset light–dark cycle (12:12 h) condition. The mice were given normal drinking water ad libitum during the experimental periods. Mice have been fed with standard pellet and water ad libitum. Mice were housed under specific pathogen-free conditions and followed the institutional guidelines concerning the care and handling animal according to Guiding Principles for the Care and Use of Animals for Scientific Purposes in the Institutional Animal Care and Use Committee (IACUC). The animal studies were performed by current regulations and ethical approval of *The Ethical Committee Brawijaya University,* Malang, Indonesia (No.405-KEP-UB).

### AOM and DSS-induced colon cancer model

**Fig. 1:**
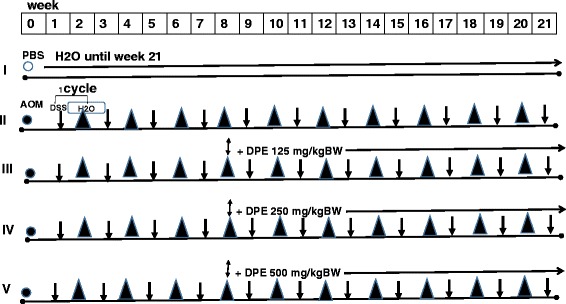
The experiment design of mice was induced Colitis Associated Colon Cancer (CAC) and DPE administration. Mouse was induced firstly by azoxymethane *(*AOM) and followed by administration of drinking water containing 5 % dextran sodium sulfate (DSS) in a cycle protocol for 7 days at weeks (1, 3, 5, 7, 9, 11, 13, 15, 17, 19 and 21) and followed by seven days of regular water at weeks (2, 4, 6, 8, 10, 12, 14, 16, 18 and 20). For the experiment, mice were divided into 5 groups; I received water (control); II was given DSS only; III-V groups were treated DPE (125 mg/kg BW, 250 mg/kg BW and 500 mg/kg BW), respectively. The administrated of DPE were started from the 8^th^ weeks until 21^th^ weeks. At the end of 21 weeks, mice in all groups were sacrificed ○ = PBS, ● = AO, ↓= DSS, ▲ = water (H_2_O), ↕ = DPE

Mice were given a single i.p. injection of 10 mg/kg azoxymethane (AOM; (Sigma-Aldrich, USA, Cat A5486) or vehicle (PBS) on experimental day 1. At one week post injection, colitis was induced by providing drinking water containing 5 % DSS (ICN Biomedical Inc, CA, USA) for a week. DSS was administered in a cycle protocol, each cycle consisting of 7 days of 5 % DSS and followed by one week of regular water. Colon cancer was induced by cyclical DSS treatment, which consisted of 1 week of 5 % DSS followed by 7 days of untreated water (Fig. 1). The oral administration of DPE starting at 8 weeks was continued until 21 weeks [21]. Mice were randomly divided into five groups (Fig. 1). Control group received water (vehicle). Group ll was given 5 % DSS only. Group (lll-V) were received DPE at 125, 250 or 500 mg/kg body weight, respectively. The dose of DPE 125 mg/kg-500 mg/kg were used previously [21]. Colon tissue was removed and cleaned, then subjected to ELISA, flowcytometry, qPCR and immunohistochemistry.

### Colon organ culture and IL-22 level assay

Colon was washed in ice-cold PBS supplemented with penicillin (100 μg/ml) and streptomycin (100 mg/ml). Then, the colons were cut into small pieces and placed into 24-well plates contain 500 ml RPMI 1640 (Sigma-Aldrich) supplemented with 10 % FBS, 100 μg/ml penicillin and 100 μg/ml streptomycin, and incubated for 24 h at 37 °C, 5 % CO_2._ IL-22 level in the supernatants were determined by ELISA kit (Biolegend, USA, Cat. 436307).

### Myeloperoxidase (MPO) activity

The activity of the enzyme myeloperoxidase (MPO) was used to assess further infiltration of neutrophils. Briefly, the colonic protein were extracted by lysing cells in 1 mM phenylmethylsulfonyl fluoride (PMSF), 10nM Tris, 200 mM NaCl, 3 mM EDTA, 10 % glycerol (pH 7,4). The extracted protein was measured for MPO levels using a mouse MPO ELISA kit according to the manufacturer’s instructions (Elabscience Cat. No:E- El - M2632).

### Immunohistochemistry for cell proliferation

The cell proliferation patterns in the colon tissue were assessed by using BrdU immunohistochemistry. We used 4 μm thick paraffin-embedded sections from the colons of the mice in all groups then visualized by BrdU incorporation. Briefly, paraffin-embedded colon tissue sections were deparaffinized and hydrated. Tris–HCl buffer (0.05 μM pH 7.6) was used to prepare solutions to rinse slides among the various steps. Incubations were performed in a humidified chamber. The sections were treated for 40 min at room temperature with 2 % bovine serum albumin and incubated overnight at 4 °C with primary antibodies mouse monoclonal anti- 5-bromo-2-deoxyuridine (BrdU) antibody (Biolegend, Cat. No: 339801) dilution of 1:200 for 1 h at room temperature. The tissue sections were sequentially incubated with biotinylated goat anti-mouse IgG (no. B0529; Sigma) and Avidin-conjugated horseradish peroxidase (Avidin Peroxidase Staining Kit, Sigma) at dilution of 1:200 and 1:30, respectively. Staining was developed with diaminobenzidine (DAB; Sigma) substrate and sections were counterstained with hematoxylin. Sections were evaluated using light microscopy and examined in a blind fashion by two individuals separately.

### Isolation and culture of mouse colon epithelial cells

The culture medium used was RPMI 1640 (Gibco) supplemented with 10 % Fetal Bovine Serum (FBS), 20 mM Hepes, 4 mM glutamine, U/ml 100 penicillin, U/ml 100 streptomycin (Sigma). Briefly, colon tissues were cut off into 1 mm pieces. This tissue fragments were incubated in HBSS (Sigma) at 37 °C under shaking for 30 min in the presence of 200 U/ml type I collagenase (Sigma) and 100 U/ml hyaluronidase (Sigma), 2 mM EDTA and 25 mM Tris (Sigma). The tissue fragments were re-suspended and incubated. The incubation procedure was repeated for two times. The supernatant containing colon epithelial cells were carrefully removed and centrifuged at 1200 rpm for 5 min at 4 °C. The supernatant containing the purified epithelial cells were collected by centrifugation at 1200 rpm at 4 °C then separated by Percoll (Sigma) density gradient.

### Cell cycle assay of epithelial cells

The cell cycle progression was assessed with BrdU followed by labeling with FITC conjugated anti-BrdU antibody and Propidium Iodide staining. Briefly, the epithelial cells were seeded in 24 well tissue culture plates (1×10^6^ cells per well). Then cells were allowed to settle for 24 h, and maintained at 37 °C in 5 % CO2 and 95 % humidity, and then the medium was replaced with 1 ml of medium containing 5 μmol BrdU. After incubation, epithelial cells were washed with phosphate buffered saline (PBS), harvested by 0.25 % trypsin, and fixed in ice-cold 70 % ethanol for 40 min. The cells were centrifuged and re-suspended nuclei in 1 ml 2 M HCl for 30 min at 37 °C. The epithelial cells were incubated in 2 ml 0.1 M sodium tetraborate (pH = 8.5) for 10 min at room temperature. Then, cells were permeabilized by 0.1 % Tween-20-1 % BSA in PBS for 5 min at room temperature, followed by staining with mouse anti BrdU (Biolegend, Cat. No:339801) in PBS and the nuclear DNA was stained with 20 μg/ml propidium iodide (PI, Sigma) at room temperature for 30 min. Finally, the epithelial cells were analyzed by using a BD FACS Calibur flow cytometer (Becton Dickinson, San Jose, CA, USA).

### RNA isolation and relative quantification by real time PCR

The level of p53 mRNA in the colon tissue was determined by real time PCR. Total RNA was extracted by using Tri Reagent according to the manufacturer’s instructions (MP Bio). according to the manufacturer’s protocol. The yield and purity of RNA was quantified by nano spectrophotometry at 260 and 280 nm. Total RNA (1 μg/ml) were reverse transcribed to to cDNA by using First Strand cDNA Synthesis (Gbioscience Cat. 786–814) according to the manufacturers. A real time assay was performed according to Light Cycler-Fast Start DNA Master^PLUS^ SYBR Green I (Roche Applied Science Light Cycler® Diagnostic, USA) Primer sequences were: p53 Forward 5′CCG ACC TAT CCT TAC CAT CATC 3′, Reverse 5′TTC TTC TGT ACG GCG GTC TTC TC 3′. β-actin Forward 5′TGG AAT CCT GTG GCA TCC ATG AAA C 3′, Reverse 5′TAA AAC GCA GCT CAG TAA CAG TCC G 3′.

The amplification program consisted of the following steps: pre-incubation at 95 °C was 10 min, followed by 40 cycles of denaturation was 95 °C was 10 s, annealing was 50 °C for 20 s and extension was 25 s. Each gene expression was normalized with β-actin mRNA content [22]. The relative induction of mRNA expression comparative Ct was calculated using ^ΔΔ^Ct was calculated using following formula: (Ct target - Ct β-actin) treatment - (Ct target - Ct β-actin) non treatment.

### Statistical analysis

The data were reported as mean ± standard deviation. The statistical significance was evaluated by using 1-way ANOVA (*p* < 0.05), followed by Post Hoc Tukey test.

## Results

### Dextran sulfate sodium induced colitis associated cancer (CAC)

Based on previous studies, DSS was used in combination with AOM, received a chemical carcinogen which induces colorectal cancer in mice. After receiving 3 cycles of DSS administration, mice received various doses group lll (doses (125 mg/kg), group lV (250 mg/kg) and group V (500 mg/kg). At the end of 21 weeks, mice in group ll (DSS only or DPE (−) had diarrhea and occult blood in feces. Blood in the feces was detected using an occult blood detection kit (Hemoccult). Body growth rate was slightly lower in the mice that received DSS administration only (Fig. 1).

### DPE decreased the level of IL-22 in the colon of CAC models

**Fig. 2:**
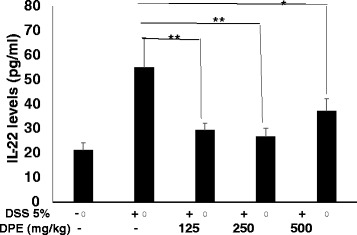
DPE decreased IL-22 levels in CAC murine models. IL-22 levels were measured from supernatants of colon organ culture by enzyme linked immunosorbent assay. Each group consisted of at least four mice. Results shown are mean + SD, with n = 4 replicates in each group. Results are mean ± SD. *, *p* < 0.05. **, *p* < 0.001

AOM and DSS administration increase IL-22 levels in supernatant colon culture significantly (*p* = 0.000). The administration of DPE at all doses in group lll (doses (125 mg/kg), *p* = 0.000; group lV (250 mg /kg), *p* = 0.000, group V (500 mg/kg) *p* = 0.010) decreased the levels of IL-22 significantly compared with DPE (−) group (Fig. 2). The greatest decrease was a dose of DPE (250 mg/kg).

### DPE decreased level of MPO

**Fig. 3:**
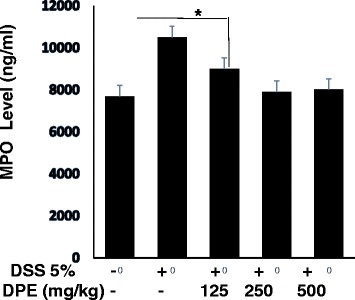
DPE decreased MPO levels in CAC murine models. The activity of the enzyme myeloperoxidase (MPO) was used to evaluate infiltration of neutrophils. MPO levels were measured from lysed colon cells. Each group consisted of at least four mice. Results shown are mean + SD, with *n* = 4 replicates in each group. Results are mean ± SD. *, *p* < 0.05. **, *p* < 0.001

The MPO level in DSS only group was higher significantly when compared with DPE treatment group (*p* = 0.02). The DSS only group showed extensive ulceration, with severe inflammatory cell infiltration. Administration of DPE caused significantly decrease MPO levels in DSS group compared to DPE ll and lll group (*p* = 0.01; *p* = 0.02) whereas treatment with DPE l group (125 mg /kg) did not affect DSS-treated mice (Fig. 3).

### DPE suppresses colonic epithelial cells proliferation

**Fig. 4:**
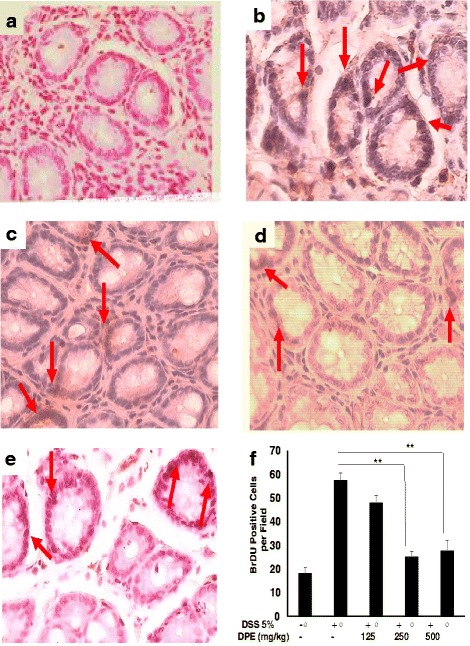
Colonic proliferation cells from the colon CAC mice models. The cell proliferation patterns in the colon tissue were assessed by using BrdU immunohistochemistry. Epithelial proliferation enhanced in colon of CAC mice model. Colon tissues were treated with anti BrDu antibody counterstained with hematoxylin and visualized under the light microscope. Cell proliferation identified by BrdU immunohistochemical staining of paraffin section. Brown-color nucleus indicates BrdU-positive cells. (A) Control group received water, (B) Group was administrated DSS 5 % only, (C), (D) and (E) mice were administrated DPE orally 125, 250 and 500 mg/kg, respectively. Red arrow: the BrdU positive cell. All images were magnified at 400x and representative four independent experiments with consistent results. (F). Number of BrdU positive cells. Results shown are mean + SD, with *n* = 4 replicates in each group. Results are mean ± SD. *, *p* < 0.05. **, *p* < 0.001

This study, epithelial cells proliferation was observed by using anti-BrdU staining performed by Immunohistochemistry. The microscopic appearance of colonic epithelial cells of CAC mice model showed undergo proliferation in DSS only group. Colonic epithelial cells proliferation was significantly higher in DSS-induced CAC models than DPE treatment groups (*p* = 0.02) (Fig. 4). Administration of DPE could decreased proliferation of epithelial cells. In this study, DPE suppressed carsinogenesis by reducing the proliferation of epithelial cells.

### DPE inhibits S phase of the cell cycle

**Fig. 5:**
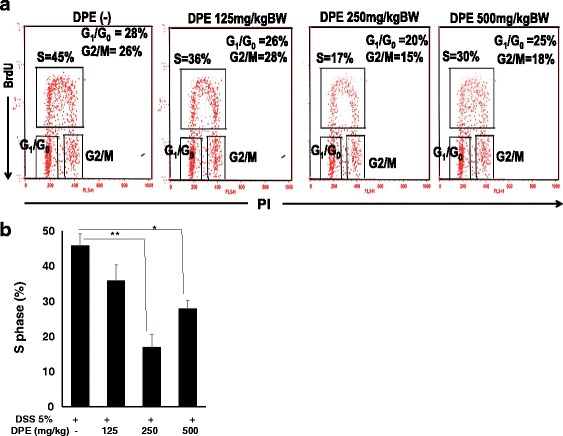
Cell cycle analysis of colonic epithelial cells treated with or without DPE. Colon Epithelial Cells 1x10^6^ cells were cultured in BrdU and the cells were cultivated for 24 h. (A). The harvest cells assessed with BrdU followed by labeling with a FITC conjugated anti-BrdU antibody and Propidium Iodide for the cell cycle were analyzed as described in Materials and Methods and the percentages of S phase, G0/G1 and G2/M in the areas have shown. Data are representative of four independent experiments with similar results. (B) The percentage of cells in S phase. Results shown are mean + SD, with *n* = 4 replicates in each group. Results are mean ± SD. *, *p* < 0.05. **, *p* < 0.001

We examined whether colonic epithelial cells were resting or proliferating state. DNA synthesis was measured in colonic epithelial cells followed. The percentage of BrdU-labeled S-phase cells were determined by observing labeled cells undergoing of the S-phase of the cell cycle. Our data demonstrated that colonic epithelial cells in CAC models without DPE treatment were predominantly in S phase (45 %), in G0/G1 phase (28 %), and in G2/M (26 %). Induction of DPE treatment change the percentage of cells in S phase through not in a dose dependent manner. DPE of 125 mg/kg BW (S phase = 36 %), 250 mg/kg BW (S phase = 17 %) and 500 mg/kg BW (S phase = 30 %) (Fig. 5).

### DPE up regulated p53 wild-type gene expression in the colon of CAC models

**Fig. 6:**
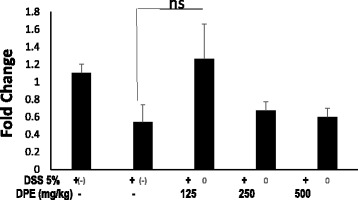
Relative gene expression of p53 from the colon of CAC models. RNA was extracted from colons, and p53 gene expression was analyzed by real time PCR and normalized by β-actin mRNA. Results are representative of four independent experiments. Results are mean ± SD. Data with statistically not significant differences (ns = not significant)

The AOM and DSS administration to Balb/C mice decreased the expression of p53 (*p* = 0.029). The expression of p53 at DSS only group decreased 49 % when compared with mice were received DPE of 125 mg/kg BW group. The expression of p53 responded to administration of DPE in a manner inversely doses dependent. The dose of DPE 125 mg group slightly increased p53 expression when it was compared with a DSS only group (*p* = 0.06). The difference was not statistically significant between DPE I 125 mg/kg, DPE II DPE 250 mg/kg and III DPE 500 mg/kg groups. Although there were slightly increased p53 expression in DPE I 125 mg/kg treatment group, the colons from CAC mice (Fig. 6).

## Discussions

In this study, we demonstrated the effect of DPE on the levels of IL-22, MPO, proliferation and wild type p53 expression in CAC mouse models. Further we analyzed the levels of IL-22 in the DSS only group were significantly higher than DPE treated groups (I and II group, (*p* < 0.001; *p* < 0.001). We have shown that administration of DPE to CAC models could decrease IL-22 and proliferation levels. DPE given to murine CAC models would be expected to decrease COX-2 and proinflammatory cytokine, which are indicators of IL-22 production [23]. Thus, in our study, DPE administration led to a significant reduction of proliferation, which was accompanied by decreased IL-22, so that it would be capable of suppressing the progression of CAC. This result is consistent with the report that *D. pentandra* extract that contain quercetin could inhibit the proliferation of a wide range of cancers such as prostate, cervical, lung, breast, and colon [24, 25].

In our study also demonstrated in DPE treated group was reduced the MPO levels. The results of the current study indicated that the ability of mice to develop CAC was inhibited by the administration of the DPE treatment. Mice were administered DPE 250 mg/kg and 500 mg/kg exhibited significantly lower MPO levels compared with untreated DPE group (*p* = 0.01; *p* = 0.00). This finding suggests that DPE treated mice were protected from inflammation. Neutrophils would amplify inflammation by secreting enzyme myeloperoxidase (MPO) to release the inflammatory mediators. It has been known that DPE contains quercetin, a compound that has function as inflammation inhibitor [20] by blocking phosphorylation inhibitors Kappa B (IKB) [3, 4, 26].

Our results show that DPE treatment causes G1 arrest of cell cycle. This study demonstrated that DPE treatment induce cell cycle inhibition in S phases. Here we show that DPE treatment impedes S-phase. This led to an examination of the molecular mechanisms of cell cycle regulation by DPE. A prominent cell cycle arrest at S phase was noted upon treatment with quercetin [27]. Besides, an reduce of cells in S phase was observed in DPE administration. This suggests that quercetin might restrain DNA synthesis. A similar G1/S phase arrest has been observed previously as well [28, 29]. Our results indicate that DPE induced p53-mediated inhibition of S phase in cell cycle.

The tumor suppressor protein (p53) plays a central role in cancer prevention and therapy. The regulation of p53 occurs in cell cycle in a variety of cells [30, 31]. In addition, DPE has up-regulated p53 function and progression at the S cell cycle phase. DPE might increase p53 expression at 125 mg/kg, indicating that DPE was able to prevented excessive cell inflammation. DPE promoted DNA synthesis as indicated by the lack presentation of S phase, and they were functionally able to suppress the development of proliferation. Meanwhile, DPE in high doses may cause increased risk of toxicity. In our study DPE was administered daily for 14 weeks. This finding suggested that the accumulation of toxic substances could effects of p53 expression. The tumor suppressor p53 as a major regulator of cell cycle progression in response to arrest of DNA synthesis [31–34]. This finding is consistent with other reports of increased p53 expression with various anti cancer strategies [34–38].

## Conclusion

In conclusion, DPE can induce cytotoxicity through multiple routes of action. Our results show that DPE could prevent proliferation by inhibition of S phase through induce p53 expression. Therefore, our results suggest that DPE is a promising candidate in cancer therapeutics.

## Abbreviations

AOM: Azoxymethane
BrdU: 5-bromo-2-deoxyuridine
CAC: Colitis associated cancer
DPE: *Dendrophtoe pentandra* extract
DSS: Dextran sodium sulfate
MPO: Myeloperoxidase
